# Bacterial profiling of White Plague Disease across corals and oceans indicates a conserved and distinct disease microbiome

**DOI:** 10.1111/mec.12638

**Published:** 2014-01-29

**Authors:** Cornelia Roder, Chatchanit Arif, Camille Daniels, Ernesto Weil, Christian R Voolstra

**Affiliations:** * Red Sea Research Center, King Abdullah University of Science and Technology23955, Thuwal, Saudi Arabia; † Department of Marine Sciences, University of Puerto RicoPO BOX 9000, Mayaguez, Puerto Rico, 00680, USA

**Keywords:** 16S rRNA gene microarray, coral disease, microbial community, *Orbicella faveolata*, *Orbicella franksi*, *Pavona duerdeni*, *Porites lutea*, White Plague Disease (WPD), White Plague-like Disease, White Syndrome (WS)

## Abstract

Coral diseases are characterized by microbial community shifts in coral mucus and tissue, but causes and consequences of these changes are vaguely understood due to the complexity and dynamics of coral-associated bacteria. We used 16S rRNA gene microarrays to assay differences in bacterial assemblages of healthy and diseased colonies displaying White Plague Disease (WPD) signs from two closely related Caribbean coral species, *Orbicella faveolata* and *Orbicella franksi*. Analysis of differentially abundant operational taxonomic units (OTUs) revealed strong differences between healthy and diseased specimens, but not between coral species. A subsequent comparison to data from two Indo-Pacific coral species (*Pavona duerdeni* and *Porites lutea*) revealed distinct microbial community patterns associated with ocean basin, coral species and health state. Coral species were clearly separated by site, but also, the relatedness of the underlying bacterial community structures resembled the phylogenetic relationship of the coral hosts. In diseased samples, bacterial richness increased and putatively opportunistic bacteria were consistently more abundant highlighting the role of opportunistic conditions in structuring microbial community patterns during disease. Our comparative analysis shows that it is possible to derive conserved bacterial footprints of diseased coral holobionts that might help in identifying key bacterial species related to the underlying etiopathology. Furthermore, our data demonstrate that similar-appearing disease phenotypes produce microbial community patterns that are consistent over coral species and oceans, irrespective of the putative underlying pathogen. Consequently, profiling coral diseases by microbial community structure over multiple coral species might allow the development of a comparative disease framework that can inform on cause and relatedness of coral diseases.

## Introduction

Corals are animals that live in a symbiotic relationship with photosynthetic dinoflagellates of the genus *Symbiodinium* as well as a rich bacterial community among other microorganisms that are collectively referred to as the coral holobiont (Rohwer *et al.* 2002). A coral's associated microbial community contributes fundamentally to the holobiont's functioning due to its role in coral nutrition (Lesser *et al.* 2004) and host defense (Ritchie & Smith 2004; Kelman *et al.* 2006; Ritchie 2006). Coral diseases are considered one of the most destructive local and geographical forces that impact corals and are responsible for major reef ecosystem declines over the past decades (Sutherland *et al.* 2004; Weil 2004; Willis *et al.* 2004; Weil *et al.* 2006; Harvell *et al.* 2007; Miller *et al.* 2009).

Coral disease is defined as any abnormal condition affecting the coral holobiont (Rosenberg *et al.* 2007), often described as a progressive loss of coral tissue due to viral, fungal, protozoan, or bacterial infections (Sutherland *et al.* 2004; Bourne *et al.* 2009) and facilitated by environmental factors (e.g. high sea surface temperatures). It usually manifests through tissue discoloration and eventually tissue loss (necrosis). While the causative agents remain unknown for most diseases (Rosenberg & Kushmaro 2011), it has been shown that compromised health in corals is accompanied by shifts in the microbial community associated with the coral holobiont (Sunagawa *et al.* 2009; Kimes *et al.* 2010; Cardenas *et al.* 2012; Cróquer *et al*. 2012; Roder *et al.* 2014). However, it is unclear whether infection of a single pathogen or opportunistic infections secondary to exposure to physiological stress trigger the restructuring of microbial communities in coral disease (Lesser *et al.* 2007). While this is mainly due to the complexity and dynamics of the host microbial assemblages (Rohwer *et al.* 2002; Hong *et al.* 2009; Littman *et al.* 2009), difficulty in conducting experiments underwater, an overall lack of information on the structure and composition of the ‘natural’ bacterial community of corals, and differences in applied methodologies further complicate the comparison of data. Sanger cloning-and-sequencing approaches are now being complemented by high-throughput methodologies and comparative analyses have shown that data from different platforms produce similar results (Sunagawa *et al.* 2009; Bayer *et al.* 2013). However, unequal sample read representation and the use of different 16S amplicon sites hinder a direct comparison between studies. PhyloChip™ 16S rRNA gene microarrays provide a standardized platform and have been successfully used to uncover microbial community patterns in coral disease (Sunagawa *et al.* 2009; Kellogg *et al.* 2012; Roder *et al.* 2014).

White Plague disease (WPD) is one of the most destructive and widespread coral diseases in Caribbean reefs (Dustan 1977; Antonius 1985; Richardson *et al.* 1998; Miller *et al.* 2009; Weil *et al.* 2009). It presents as a bright white band (i.e. clean skeletal structure resulting from disappearing tissue) that initiates at the base or sides of a colony and separates the living tissue from recently settled turf algae on the exposed skeleton that quickly advances across the colony surface. Depending on the type of WPD (I, II or III), progression rates vary and different coral species are affected (Sutherland *et al.* 2004; Weil & Rogers 2011). *Aurantimonas coralicida* (Denner 2003) and *Thalassomonas loyana* (Thompson *et al.* 2006) were proposed as causative agents of WPD or WPD-like in corals from the Caribbean and the Red Sea, respectively. However, subsequent studies were unable to detect either of these two putative pathogens (Pantos *et al.* 2003; Barash *et al.* 2005; Sunagawa *et al.* 2009; Cardenas *et al.* 2012; Roder *et al.* 2014) suggesting that different pathogens must be able to produce highly similar disease phenotypes (Weil 2004; Reshef *et al.* 2008; Weil & Rogers 2011). In the Great Barrier Reef and Indo-Pacific region, phenotypes of WPD-like etiopathology have been denominated White Syndrome (WS) (Willis *et al.* 2004), and strains of the coral-bleaching pathogen *Vibrio coralliilyticus* have been identified as potential infectious agents in a number of coral species (Sussman *et al.* 2008). Accordingly, indistinguishable disease phenotypes are produced by different pathogens (Weil & Rogers 2011), and disease nomenclature can be mistaken. For this reason, we refer to coral colonies displaying visual characteristics of White Syndrome, White Plague or White Plague-like disease as WPD, acknowledging that this neither includes nor excludes the presence of the pathogens *A. coralicida*, *T. loyana* or *V. coralliilyticus*. On the other hand, how this convergent phenotypic resemblance relates to similarities in shifts in the underlying microbial community structure is at present unknown.

In this study, we analysed microbial communities of healthy and WPD-affected coral tissues of *Orbicella faveolata* and *Orbicella franksi* (former genus *Montastraea,* Budd *et al*. (2012)) from the Caribbean (Puerto Rico). Subsequently, we compared these data to microbial communities of two coral species, *Pavona duerdeni* and *Porites lutea*, displaying WPD characteristics (Dustan 1977; Richardson 1998) from the Indo-Pacific (Gulf of Thailand) (Roder *et al.* 2014). We employed 16S rRNA gene microarrays (PhyloChips™) assaying 59 222 operational taxonomic units (OTUs) to profile microbial communities of healthy (HH) and diseased (DD) coral colonies in a standardized framework. We aimed to determine whether microbial community patterns of healthy and diseased colonies are not only consistent between species from the same site (Roder *et al.* 2014), but also over larger geographical distances, and how these patterns change between closely related and more distantly related coral species.

## Material and methods

### Study site and sample collection

Sampling took place on 5 and 6 September 2011 at Weimberg reef (between N 17°53′17.40/W 66°59′52.90 and N 17°53′25.40/W 66°59′19.00) off the southwest coast of Puerto Rico. Two coral species (*Orbicella faveolata* and *O. franksi*) were sampled via SCUBA between 16 and 22 m depth. From both species, tissue samples from three healthy colonies (displaying no visible signs of stress) and three colonies with WPD phenotype were collected. All corals were of similar size. All healthy samples were collected from the upper, nonshaded surface of the coral colony using hammer and chisel and were immediately transferred to sterile Whirl-pak bags. Samples of corals displaying signs of WPD were taken directly from the interface of healthy and diseased tissue. All samples were kept on ice during transportation.

### Sample processing and data generation

Upon return to the laboratory, samples were rinsed with filtered seawater (0.22 μm) to remove loosely associated microbes. Rinsed samples were subsequently flash-frozen in liquid nitrogen and ground to powder using mortar and pestle. Samples were then processed as described in Roder *et al*. (2014). Briefly, DNA was extracted from the coral powder using the DNeasy Plant Kit (Qiagen, Hilden, Germany). After quantification of DNA using a NanoDrop 2000C spectrophotometer (Thermo Fisher Scientific, Waltham, MA, USA) and a Qubit fluorometer (Quant-IT dsDNA Broad Range Assay Kit; Invitrogen, Carlsbad, CA, USA), DNA was shipped on dry ice to Second Genome Inc. (San Bruno, CA, USA) for hybridization to the PhyloChip™ G3 platform as described in Hazen *et al*. (2010). Up to 500 ng of PCR product was applied to each PhyloChip™ G3 following previously described procedures (Hazen *et al.* 2010). Hybridized arrays were washed, stained, and scanned as previously described (Hazen *et al.* 2010). Array fluorescence intensities (HybScores) (Table S1, Supporting information) were Loess-normalized using the normalize.loess function in the affy package (Gautier *et al.* 2004) in the R statistical environment (R Development Core Team 2008) to obtain abundance data for OTUs present in *O. faveolata* and *O. franksi* (*n *= 11 256 OTUs). A microbial taxon was regarded present if it was identified in two of three replicates of any species/condition combination (*O. faveolata* HH, *O. faveolata* DD, *O. franksi* HH, *O. franksi* DD) (Table S2, Supporting information). Comparisons of these data to PhyloChip™ results from healthy and WPD-affected samples of *Porites lutea* and *Pavona duerdeni* collected in the Indo-Pacific (Roder *et al.* 2014) were made on Loess-normalized HybScores of shared OTUs (*n *= 7200 OTUs). An OTU was considered present if it was detected in two of three replicates for any species/condition combination (i.e. *O. faveolata* HH, *O. faveolata* DD, *O. franksi* HH, *O. franksi* DD, *P*. *lutea* HH, *P. lutea* DD, *P. duerdeni* HH, *P. duerdeni* DD).

### Data analysis

We calculated OTU richness for the total data set as well as for any species/condition combination. To derive total richness of both data sets, that is, from the Caribbean (this study) and the Indo-Pacific (Roder *et al.* 2014), the amount of all OTUs present was determined, and OTUs shared between both, unique to either data set, or shared between conditions were compared.

Statistical evaluation of the Caribbean data set included all bacterial OTUs present (*n *= 11 256 OTUs). First, differentially abundant OTUs between species (*O. faveolata* vs. *O. franksi*), condition (HH vs. DD), and their interactions were determined based on normalized HybScores applying a two-way factorial analysis of variance (anova) (Table S3, Supporting information) using the TM4 software (Saeed *et al.* 2003). *P*-values were adjusted applying a 10% False Discovery Rate (FDR) using the QVALUE software package in R (Storey 2002). Log2 fold-changes in OTU abundance between different conditions were derived by averaging the abundance estimates (i.e. HybScores) for the 3 replicates and subsequent subtraction of HH from DD and division by 1000. Note that HybScores are log_2_ transformed fluorescence intensity values ranging from 1 to 65 536 (2^0^–2^16^) that are multiplied by 1000 yielding a range of 0–16 000.

Relationships between samples from the two combined data sets (Caribbean and Indo-Pacific) were illustrated using multidimensional scaling (MDS) based on Bray–Curtis distances between samples using MASS and vegan library in R (R Development Core Team 2008), in which the stress value depicts the accuracy of the ordination. Effects of the three factors ‘site’ (Caribbean vs. Indo-Pacific), ‘species’ (*P. duerdeni* vs. *P. lutea* vs. *O. faveolata* vs. *O. franksi*) and ‘condition’ (HH vs. DD) were calculated using the permutational multivariate analysis of variance (PERMANOVA) add-on in PRIMER-E (Anderson 2005). In the PERMANOVA design, we nested the factor ‘species’ within the factor ‘site’, as different species pairs were sampled at the two study sites. 999 permutations (only 998 unique for ‘condition’, only 997 unique for interaction term ‘site’×‘condition’ and only 997 unique for interaction term ‘condition’×‘species(site)’) of residuals under a reduced model were conducted (Anderson & Legendre 1999; Anderson & ter Braak 2003). The resulting Pseudo-F and associated *P*-values can be interpreted as equal to results of univariate anovas, however, are based on the multivariate Bray–Curtis distance measures (Anderson 2005).

Differentially abundant OTUs for the combined data from the Caribbean and Indo-Pacific were determined via two-way anova, as described for the Caribbean samples above. We classified those OTUs that were more than 2-fold differentially abundant in the same direction (HH or DD) in all four species (*P. duerdeni*, *P. lutea*, *O. faveolata*, *O. franksi*) as footprint bacterial species in WPD. These respective microbial key players were additionally sorted according to their respective families. Finally, we compared phylogenetic relationships of the coral species to differences in bacterial assemblages (*n *= 7200 OTUs) in HH samples. Phylogenetic relationships between the four coral species (*O. faveolata*, *O. franksi*, *P. lutea* and *P. duerdeni*) (Romano & Palumbi 1996; Fukami *et al.* 2008; Budd *et al.* 2012) were compared to dendrograms based on similarities in bacterial assemblages. Dendrograms were constructed by averaging normalized OTU HybScores over samples for all HH species and subsequent application of Euclidean distance clustering (average linkage) with 1000 bootstraps using the TM4 software (Saeed *et al.* 2003). Trees were visualized using TreeView (Page 1996).

## Results

### Bacterial richness in healthy and diseased corals

Of the 59 222 microbial OTUs assayed on the PhyloChip™ G3 microarray, 11 256 OTUs were present in the coral samples collected from the Caribbean. OTU numbers were similar for both species (*Orbicella faveolata* and *O. franksi*) with more than a 50% increase in OTU richness in diseased corals compared with healthy specimens (Table 1). The numbers of detected OTUs were similar to results from a previous study in the Indo-Pacific, where we assayed healthy and WPD-affected colonies in *Pavona duerdeni* and *Porites lutea* (Roder *et al.* 2014). In both surveys, we observed increased richness in DD samples compared with HH. Combining OTU richness from both studies, we found a total of 18 269 distinct OTUs over the four different coral species. Of these, around 40% (7200 OTUs) were shared between coral species from both regions, while the remaining OTUs were distributed unevenly between corals from the Caribbean (4056 nonshared OTUs) and the Indo-Pacific (7013 nonshared OTUs). Of all OTUs present in either study, more than three times as many OTUs (7122) were found in diseased samples in comparison with OTUs from healthy samples (2335). *Aurantimonas coralicida* or *Thalassomonas loyana*, the two proposed causative agents of WPD, were not detected in any sample from both data sets analysed with the PhyloChip™ platform. Further, strains of *Vibrio coralliilyticus*, identified as proposed WS pathogens by Sussman *et al*. (2008), were not represented on the PhyloChip™ platform, and accordingly, not assayed.

**Table 1:** OTU richness from PhyloChip™ hybridizations investigating WPD in four species from the Caribbean (Puerto Rico) and the Indo-Pacific (Gulf of Thailand)

PhyloChip	# Bacterial OTUs
This study – Puerto Rico, Caribbean	
Total	11 256
In *Orbicella faveolata* HH	4336
In *Orbicella* *faveolata* DD	6791
In *Orbicella franksi* HH	4538
In *Orbicella franksi* DD	7448
Roder *et al*. (2014) – Gulf of Thailand, Indo-Pacific	
Total	14 213
In *Pavona duerdeni* HH	2756
In *Pavona duerenii* DD	4434
In *Porites lutea* HH	7580
In *Porites lutea* DD	10 848
Both studies	
Total	18 269
Unique Puerto Rico (Caribbean)	4056
Unique Gulf of Thailand (Indo-Pacific)	7013
Shared (Puerto Rico and Gulf of Thailand)	7200
Associated with HH (Caribbean and Indo-Pacific)	2335
Associated with DD (Caribbean and Indo-Pacific)	7122

OTU richness is shown for all four species/condition combinations from the Caribbean (*O. faveolata* HH, O*. faveolata* DD, *O. franksi* HH, *O. franksi* DD) and from the Indo-Pacific (*P. duerdeni* HH, *P. duerdeni* DD, *P. lutea* HH, *P. lutea* DD). Bacterial taxa were counted present when detected in 2 of 3 replicates of any species/condition combination.

### Differentially abundant OTUs in healthy and diseased corals from the Caribbean

A two-way anova was conducted to test for significant differences in OTU abundances between conditions (HH vs. DD), species (O*. faveolata* vs. *O. franksi*) and combinations thereof (Table 2). While no bacterial taxon differed significantly in abundance between the two coral species, 2411 OTUs were significantly different between HH and DD samples. Log2 fold-changes (FC) in bacterial abundance ranged from −5.03 (OTU 72172, genus Fusobacterium: ∼32-fold higher in HH) to +3.10 (OTU 61563, family Rhodobacteraceae: ∼8-fold higher in DD) in *O. faveolata* (Table S3, Supporting information). In *O. franksi*, log2 bacterial abundance changes ranged from −6.29 (OTU 76854, genus Spirochaeta: >70-fold higher in HH) to +5.08 (OTU 51567, phylum Acidobacteria, class PAUC37f: ∼30-fold higher in DD) (Table S3, Supporting information). Average log2 fold-changes were higher in *O. franksi* (log_2_FC = 2.42 ± 0.41) in comparison with *O. faveolata* (log_2_FC = 1.21 ± 0.70).

**Table 2:** Summary statistics of two-way anova separating species and condition effects between *Orbicella faveolata* and *Orbicella franksi*

Two-way anova (11 256 OTUs, FDR <0.1)	# Bacterial OTUs
Species significant (*O*. *franksi* vs. *O. faveolata*)	0
Condition significant (HH vs. DD)	2411
Interaction significant: species × condition	1

Data based on normalized HybScores of 11 256 OTUs present over all coral samples.

Approximately half of the OTUs significantly different between HH and DD samples were more abundant in HH compared with DD for both *Orbicella* species, about 40% were more abundant in DD, and <10% of the bacterial OTUs either increased or decreased when assessing condition-specific OTUs per coral species (Table S3, Supporting information). Only a single bacterial taxon, *Jannaschia* sp.*,* was found to differ significantly in abundance in a species-and-condition-type interaction confirming that bacterial abundance patterns in healthy and diseased corals seem to be conserved and distinct from coral species-specific bacteria for the majority of OTUs analysed, as found by Roder *et al*. (2014).

### Comparison of bacterial patterns in WPD from the Caribbean and the Indo-Pacific

**Figure 1:**
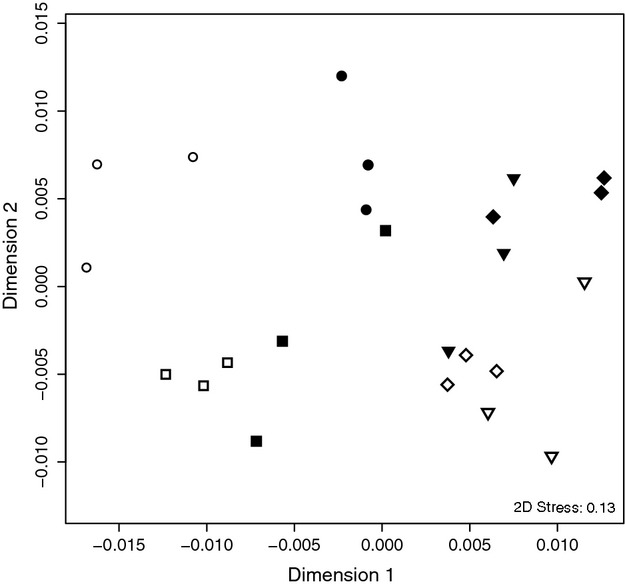
Similarities between coral species, health state and ocean basin based on microbial communities. Multidimensional scaling (MDS) plot derived from Bray–Curtis distances of normalized PhyloChip™ HybScores (*n *= 7200 OTUs). Healthy (open) and WPD-affected (filled) specimens of the corals *P. duerderni* (square), *Porites lutea* (circle), *Orbicella faveolata* (triangle) and *Orbicella franksi* (diamond) are shown. Stress represents the goodness of fit of the data onto the MD ordination.

We combined data from this study with data from a previous study in the Indo-Pacific (Roder *et al.* 2014) to compare bacterial abundance patterns in healthy and diseased corals across oceans (i.e. the Caribbean and the Indo-Pacific) and coral species (i.e. *O. franksi*, *O. faveolata*, *P. duerdeni*, *P. lutea*). For this analysis, we only considered OTUs present in both data sets (*n *= 7200). Relationships based on OTU diversity of the samples were displayed in an MDS ordination (Fig. 1), and the relative contributions of ‘site’, ‘species’, and ‘condition’ were tested via PERMANOVA (Table 3). While all factors contributed highly significant to the structure of the data (*P *<* *0.001), the denominator ‘site’ was the most pronounced factor as indicated by the highest Pseudo-F value (Pseudo-F = 10.40). The contribution of the factor ‘site’ was more than twice as high as that of ‘condition’ (Pseudo-F = 4.90) or ‘species’ (Pseudo-F = 2.82). As the combined data sets did not comprise the same coral species in both regions, the factor ‘species’ was nested within the factor ‘site’ and therefore yielded a lower Pseudo-F, even though the sum of squares was higher than for the factor ‘condition’ (20.72 vs. 18.02). Furthermore, interaction terms of ‘site’ and ‘condition’ were significant, while there was no significant interaction between the factors ‘condition’ and ‘species’. This pattern recaptures what we saw when analysing diseased corals from the Indo-Pacific: a strong separation by condition and a slightly stronger separation by species, but no interaction between species and condition (Roder *et al.* 2014). PERMANOVA results were corroborated by the MDS plot where the largest separation was between the two sites (i.e. HH samples of either ocean were distributed towards the edges of the plane), while all DD samples were arranged amid the HH samples. This indicates that bacterial communities associated with different corals, irrespective of species or site, are more similar when affected by WPD than when corals are healthy (Fig. 1).

**Table 3:** Factors influencing bacterial communities of healthy and WPD-affected corals

	df	SS	MS	Pseudo-F	*P*_perm_	Permutations
Site	1	38.28	38.28	10.40	0.001	999
Condition	1	18.02	18.02	4.90	0.001	998
Species (site)	2	20.72	10.36	2.82	0.001	999
Site × condition	1	11.33	11.33	3.08	0.002	997
Condition × species (site)	2	10.28	5.14	1.40	n.s.	997
Residuals	16	58.87	3.68			
Total	23	157.50				

PERMANOVA summary statistics for factors affecting bacterial communities in healthy and WPD-affected corals from the Caribbean (i.e. Puerto Rico) and the Indo-Pacific (i.e. Gulf of Thailand).

Df, degrees of freedom; SS, sum of squares; MS, mean squares; n.s., not significant.

**Figure 2:**
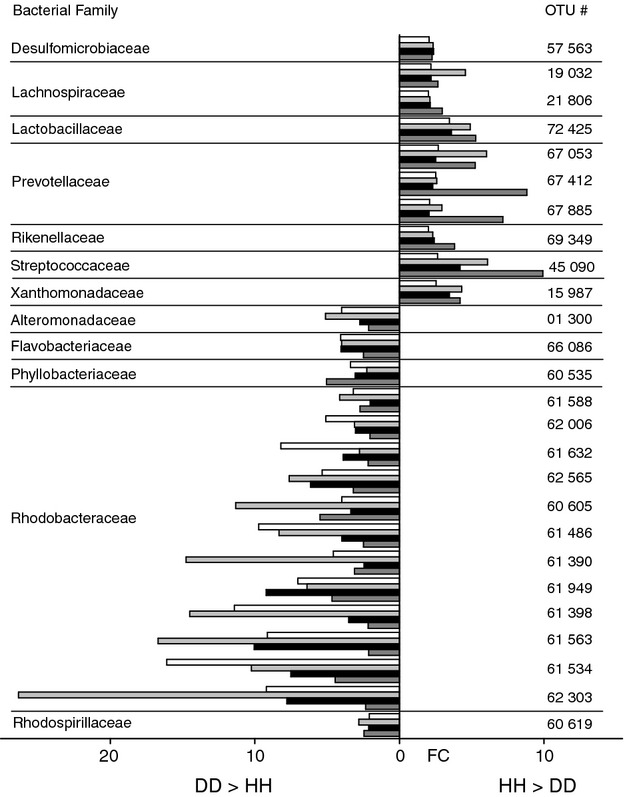
Bacterial footprints of WPD. Displayed are bacterial families and OTUs that showed a >2-fold abundance difference between HH and DD over samples from *Pavona duerdeni* (white), *Porites lutea* (light grey), *Orbicella faveolata* (black) and *Orbicella franksi* (dark grey). FC, fold change.

To identify key bacterial species and community shifts (i.e. bacterial footprints) within the microbiomes of healthy and diseased corals, we conducted a two-way anova and determined all those OTUs that were more than 2-fold different between HH and DD in all four species and assorted them to bacterial families (Fig. 2). For those taxa >2-fold more abundant in HH corals, only 2 bacterial families (Lachnospiraceae, Prevotellaceae) were represented by more than 1 OTU, whereas 5 bacterial families (Desulfomicrobiaceae, Lactobacillaceae, Rikenellaceae, Streptococcaceae, Xanthomonadaceae) were each represented by 1 OTU. Except for *Desulfomicrobium orale* (OTU 57563), none of the OTUs could be assigned to a described bacterial species. Those bacterial taxa with more >2-fold higher abundances in DD corals were comprised of 4 OTUs from 4 families (Alteromonadaceae, Flavobacteriaceae, Phyllobacteriaceae, Rhodospirillaceae) and 12 OTUs belonging to the family Rhodobacteraceae. Accordingly, the family Rhodobacteraceae contributed 75% of the key OTUs in DD samples and also represented the highest fold-changes. The only OTU assigned to the species level from this bacterial family was *Rhodobacter sphaeroides* (OTU 61632), while all other bacterial taxa were taxonomically unclassified at the species level.

### Phylogenetic position of the coral host and bacterial community structure

**Figure 3:**
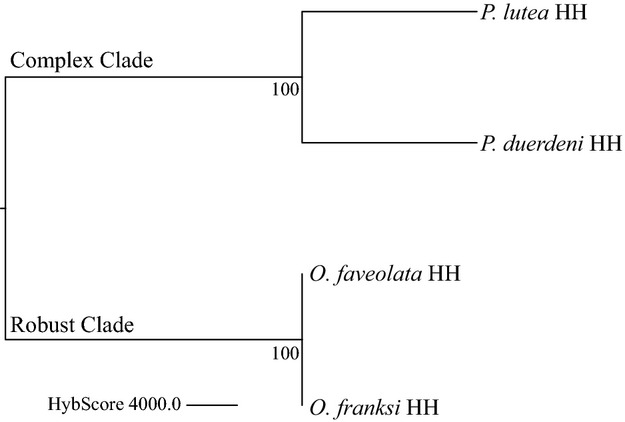
Grouping of corals based on underlying bacterial community structures. Relationship between healthy coral species in regard to differences in their bacterial community based on Euclidean distance of bacterial abundance (i.e. HybScores) from 7200 shared OTUs (1000 bootstraps). The dendrogram recaptures the phylogenetic relationship of the corals *Orbicella franksi*, *Orbicella faveolata*, *Pavona duerdeni* and *Porites lutea*.

While the main factor structuring all eight species/condition combinations appeared to be region, bacterial community profiles of DD samples were closely related to each other irrespective of species or site (Fig. 1). To further understand the contribution of phylogenetic positioning of a coral species to its microbial abundance pattern, HH samples were clustered based on microbial diversity and compared to the phylogeny between the four coral species (Fig. 3). Samples from healthy colonies recaptured phylogenetic positioning of the coral species. Furthermore, similarity between corals decreased with increasing phylogenetic distance, that is, healthy specimens of the two closely related *Orbicella* species harboured a more similar microbial abundance pattern than the more distantly related *P. duerdeni* and *P. lutea*. The greatest difference between HH bacterial abundance patterns of all four species was between complex and robust corals that are evolutionarily separated by >200 million years (Romano & Palumbi 1996). However, species pairs from a given site in our study (i.e. O*. franksi* and O*. faveolata* from the Caribbean, *P. duerdeni* and *P. lutea* from the Indo-Pacific) coincided with affiliation to the robust (*O. franksi* and *O. faveolata*) and complex (*P. duerdeni* and *P. lutea*) clades, so we were not able to disentangle whether the differences arose from phylogenetic or ocean basin (site) affiliation.

## Discussion

### Bacterial richness in healthy and diseased corals

The numbers of OTUs detected in samples from the Caribbean were comparable to those detected in other studies applying PhyloChips™ in corals (Sunagawa *et al.* 2009; Kellogg *et al.* 2012; Roder *et al.* 2014), but higher than those in sequencing-based surveys (e.g. Cardenas *et al.* 2012). Richness and diversity of bacterial taxa associated with two *Orbicella* species were similar within the same condition (HH and DD) as has been shown for microbial communities of closely related sponge species (Schoettner *et al.* 2013). We also found higher numbers of bacterial taxa associated with diseased samples, which concurs with previous studies on coral disease (Pantos *et al.* 2003; Sunagawa *et al.* 2009; Cróquer *et al*. 2012; Roder *et al.* 2014). This is suggested to be a consequence of bacterial colonization from the surrounding environment on compromised and vulnerable coral tissues (Lesser *et al.* 2007; Sunagawa *et al.* 2009; Roder *et al.* 2014) and supported in this work by a larger number of shared OTUs, and an increase in richness of unclassified bacteria in DD samples. It is worthwhile noting that bacterial diversity has been reported to be similar and overlapping across different oceans (Martiny *et al.* 2006). Accordingly, colonization of bacteria from the surrounding water column would result in similar microbial profiles of diseased tissue even between geographically distinct regions.

### Towards elucidation of bacterial disease footprints and microbiomes

A great advantage of the PhyloChip™ microarray is that it represents a standardized platform in which data over different studies can be easily integrated, comprehensively analysed and compared in a common framework. While our recent analysis of two coral species from the same reef in the Indo-Pacific indicated that microbial community patterns of health and disease are conserved over coral species boundaries (Roder *et al.* 2014), we were not able to test whether these patterns hold true over geographical distances (i.e. regionally and globally) in coral species. The experimental design of this study matches and complements that of Roder *et al*. (2014), enabling us to integrate and compare healthy and diseased states of four coral species (*Pavona duerdeni*, *Porites lutea*, *Orbicella faveolata*, *Orbicella franksi*) comprising three genera (*Pavona*, *Porites*, *Orbicella*) from two distinct regions (Caribbean and Indo-Pacific). Most interestingly, almost half of all the bacterial taxa found in either data set were shared (*n *= 7200 OTUs), even though we analysed different coral species and different regions at different depths. We found that region is the strongest separating factor between the two data sets. However, this measure took inadvertently the closer phylogenetic relationship between *O. faveolata* and *O. franksi* into account, so that we were not able to differentiate between similarity due to common reef of origin and similarity due to phylogenetic relatedness. Profiling bacterial diversity in healthy and diseased tissues from the same coral species over geographical distances will unequivocally resolve the relative contribution of phylogenetic similarity vs. health condition (e.g. data from this study and Kellogg *et al*. (2013)).

Between closely related coral species (i.e. *O. faveolata* and *O. franksi*), microbial abundance patterns were indistinguishable (as indicated by no significant different OTUs between species in the two-way anova) and support the notion that there is a close relationship between a coral host and its bacterial assemblage (Sunagawa *et al.* 2010). Indeed, grouping of corals based on bacterial community structure of HH specimens (Fig. 3) indicates that microbial assemblage resembles phylogenetic position of the coral host. Besides, diseased corals were more similar to each other than to healthy ones as indicated by the MDS plot (Fig. 1). Last, we detected no bacteria in the data set from the Indo-Pacific (Roder *et al.* 2014) and only one OTU in the data set from the Caribbean (this study) that were significantly different between species and at the same time significantly different between conditions. These data substantiate the previous notion that shared disease-specific microbial community patterns exist that are distinct form nonshared species-specific bacterial assemblages (Roder *et al.* 2014).

Bacterial taxa that were consistently found in WPD-affected coral species analysed here comprised only few families and support the hypothesis of a bacterial abundance pattern in WPD that is structured by opportunists. For instance, Pelagibacteraceae are abundant in ocean surface bacterioplanktion communities (Morris *et al.* 2002), and Rhodospirillaceae have been reported to become abundant in heterotrophic environments (Landa *et al.* 2013). Most notable, OTUs from the family Rhodobacteraceae made up 75% of all OTUs more than 2-fold enriched in DD, and fold-changes were more drastic than for any other bacterial family. As none of the members of the Rhodobacteraceae family are known to be potentially pathogenic, we hypothesize that the reason for their colonization success must either lie in the specific characteristics of this family (e.g. competence for rapid proliferation) or be due to prevailing environmental conditions (e.g. high abundance in environment). Many members of the Rhodobacteraceae are photoheterotrophs, and their ability to accumulate in response to availability of organic substrate (Landa *et al.* 2013) might present optimal conditions for effective growth in or on compromised coral tissues. Accumulation of opportunistic bacteria has been suggested to result in phenotypically similar mortality patterns (diseases) that might not necessarily have the same cause (Lesser *et al.* 2007). WS, WPD or WPD-like infections have been reported for a variety of coral species and regions (Richardson 1998; Sutherland *et al.* 2004; Willis *et al.* 2004; Bourne *et al.* 2009; Kellogg *et al.* 2013), but only three bacterial species have been proposed as causative agents (Denner 2003; Thompson *et al.* 2006; Sussman *et al.* 2008). Also, rates of the disease's progression have been shown to be different (Dustan 1977; Richardson *et al.* 1998, 2001). Conversely, while it is likely that distinct pathogens might cause WPD in different coral species, similarity in phenotypes and etiopathologies might be driven by opportunistic bacteria that are conserved over species boundaries and that structure microbial abundance patterns.

Taken together, our data indicate that (i) disease-specific microbial abundance patterns exist, which are (ii) conserved across coral species and oceans, and which are (iii) largely different from species-specific abundance patterns. While the increase in bacterial diversity from WPD specimens over ocean boundaries renders the hypothesis of colonization of opportunistic bacteria a likely scenario, the trigger of opportunistic colonization remains unknown and might well be of varying origin and importance. Conserved microbial community patterns provide the opportunity to derive bacterial families and species whose relative abundance can serve as indicators for health, stress and disease. The elucidation of commonalities in coral diseases beyond species and sites would allow the establishment of ‘bacterial footprints’, that is, shared community profiles for a given disease that can inform on the state of the coral holobiont irrespective of the coral species and/or region under study. Furthermore, such bacterial footprints would allow placing different coral diseases into a comparative framework and specifically test evolutionary hypotheses in regard to cause, origin and relatedness of different coral diseases.

## Data accessibility

PhyloChip abundance data, OTU presence/absence data, anova data: uploaded as online supporting information.

## Supporting Information

Table S1 HybScores for all present OTUs (*n *= 11 256) of PhyloChip™ microarrays from *Orbicella faveolata* HH, DD and *Orbicella franksi* HH, DD.

Table S2 Presence/Absence calls of 11 256 OTUs over all samples on PhyloChip™ microarrays (0 = absent, 1 = present).

Table S3 Two-way anova on 11 256 OTUs that were detected present in healthy and diseased specimens of the corals *Orbicella faveolata* and *Orbicella franksi*.
